# Design of high-oleic tobacco (*Nicotiana tabacum* L.) seed oil by CRISPR-Cas9-mediated knockout of *NtFAD2–2*

**DOI:** 10.1186/s12870-020-02441-0

**Published:** 2020-05-25

**Authors:** Yinshuai Tian, Kai Chen, Xiao Li, Yunpu Zheng, Fang Chen

**Affiliations:** 1 College of Landscape and Ecological Engineering, Hebei University of Engineering, No.19 Taiji Road, Economic and technological development area, Handan, 056038 Hebei China; 2 Institute of New Energy and Low-carbon Technology, Sichuan University, Chuanda Road, Shuangliu district, Chengdu, 610207 Sichuan China; 3 Key Laboratory of Bio-resources and Eco-environment of Ministry of Education, College of Life Sciences, Sichuan University, No.29 Wangjiang Road, Wuhou district, Chengdu, 610065 Sichuan China; 4 School of Water Conservancy and Hydroelectric Power, Hebei University of Engineering, No.19 Taiji Road, Economic and technological development area, Handan, 056038 Hebei China

**Keywords:** *Nicotiana tabacum*, Tobacco seed oil, CRISPR-Cas9, *FAD2*, High oleic content, Biodiesel

## Abstract

**Background:**

Tobacco seed oil could be used as an appropriate feedstock for biodiesel production. However, the high linoleic acid content of tobacco seed oil makes it susceptible to oxidation. Altering the fatty acid profile by increasing the content of oleic acid could improve the properties of biodiesel produced from tobacco seed oil.

**Results:**

Four *FAD2* genes, *NtFAD2–1a*, *NtFAD2–1b*, *NtFAD2–2a*, and *NtFAD2–2b*, were identified in allotetraploid tobacco genome. Phylogenetic analysis of protein sequences showed that *NtFAD2–1a* and *NtFAD2–2a* originated from *N. tomentosiformis*, while *NtFAD2–1b* and *NtFAD2–2b* from *N. sylvestris*. Expression analysis revealed that *NtFAD2–2a* and *NtFAD2–2b* transcripts were more abundant in developing seeds than in other tissues, while *NtFAD2–1a* and *NtFAD2–1b* showed low transcript levels in developing seed. Phylogenic analysis showed that *NtFAD2–2a* and *NtFAD2–2b* were seed-type *FAD2* genes. Heterologous expression in yeast cells demonstrated that both NtFAD2–2a and NtFAD2–2b protein could introduce a double bond at the Δ^12^ position of fatty acid chain. The fatty acid profile analysis of tobacco *fad2–2* mutant seeds derived from CRISPR-Cas9 edited plants showed dramatic increase of oleic acid content from 11% to over 79%, whereas linoleic acid decreased from 72 to 7%. In addition, the fatty acid composition of leaf was not affected in *fad2–2* mutant plants.

**Conclusion:**

Our data showed that knockout of seed-type *FAD2* genes in tobacco could significantly increase the oleic acid content in seed oil. This research suggests that CRISPR-Cas9 system offers a rapid and highly efficient method in the tobacco seed lipid engineering programs.

## Background

Tobacco (*Nicotiana tabacum* L.) is cultured worldwide as an industrial crop traditionally used for manufacturing cigarettes. Recently, tobacco seed oil had been demonstrated to be an appropriate feedstock for biodiesel production [1–4]. Tobacco is an oilseed plant with oil content ranging from 36 to 41% of seed dry weight [5, 6]. Fatty acid composition of tobacco seed oil shows a main presence of palmitic acid (8.83–12.3%), stearic acid (2.1–3.3%), oleic acid (9.97–11.69%), and linoleic acid (64.38–75.9%) [1, 7, 8]. Feedstock supply is one of the main limiting factors for biodiesel production. A high seed yield tobacco variety (Solaris) had been generated for seed oil production [9]. Life cycle analysis indicates that the production of Solaris tobacco biodiesel creates impacts that are similar to those that have been identified in biodiesel production from other crops [10]. In addition, the fuel properties of biodiesel, including cold-temperature flow characteristics, oxidative stability and NOx emissions, are other factors limiting the wide apply of biodiesel. Fuel properties of biodiesel are mostly dependent on the fatty acid composition of plant seed oil [11]. Thus, altering the fatty acid profile could improve fuel properties of biodiesel.

The high linoleic acid content makes biodiesel produced from tobacco seed oil more susceptible to oxidation, which would limit its use in traditional engine. The microsomal Δ^12^ oleate desaturase (1-acyl-2-oleoyl-sn-glycero-3-phosphocholine Δ^12^ desaturase or FAD2) desaturates oleic acid to linoleic acid in the ER. In the past few decades, *FAD2* genes from a number of plant species had been cloned and characterized [12–18]. Previous study reported that suppression of the tobacco *NtFAD2* gene by RNAi (RNA interference) could significantly increase the oleic acid content. Same strategy had been adopted to increase the oleic acid content in other oil seed crops, such as soybean [19], *Brassica rapa* [20], cotton [21], and peanut [22]. In addition, artificial microRNAs [23], TALENs (transcription activator-like effector nucleases) [24] and standard mutagenesis [25–28] methods were also used for *FAD2* gene suppression.

Although RNAi and artificial microRNA are useful tools for gene functional characterization, the continuously inheritance requirement for the T-DNA construct limited the application of these technologies in plant genetic breeding [29]. Recently, CRISPR (clustered regularly interspaced short palindromic repeats)-Cas9 system has emerged as a robust and versatile tool for genome editing in a variety of organisms [30–32]. Critically different from RNAi and artificial microRNA technologies, which generate knock-down by repressing transcripts of the targeted genes, CRISPR-Cas9 system could produce defined gene knockout by altering the genomic DNA sequence [33, 34]. Most recently, CRISPR-Cas9 system had been successfully adopted to produce high oleic seed oil by knockout *FAD2* genes in a variety of crop species, including *Camelina sativa* [35], peanut [36], *Brassica napus* [29], and soybean [37]. In this paper, we identified four *FAD2* genes in tobacco genome. CRISPR-Cas9 vector was designed to specifically knockout the seed-type *NtFAD2–2a* and *NtFAD2–2b* genes. The fatty acid profile of the tobacco mutant seeds showed a high oleic acid and low linoleic acid phenotype. The high oleic acid mutant lines generated in this paper could be used as a valuable material for further seed lipid engineering.

## Results

### Four *FAD2* genes were identified in tobacco genome

**Fig. 1:**
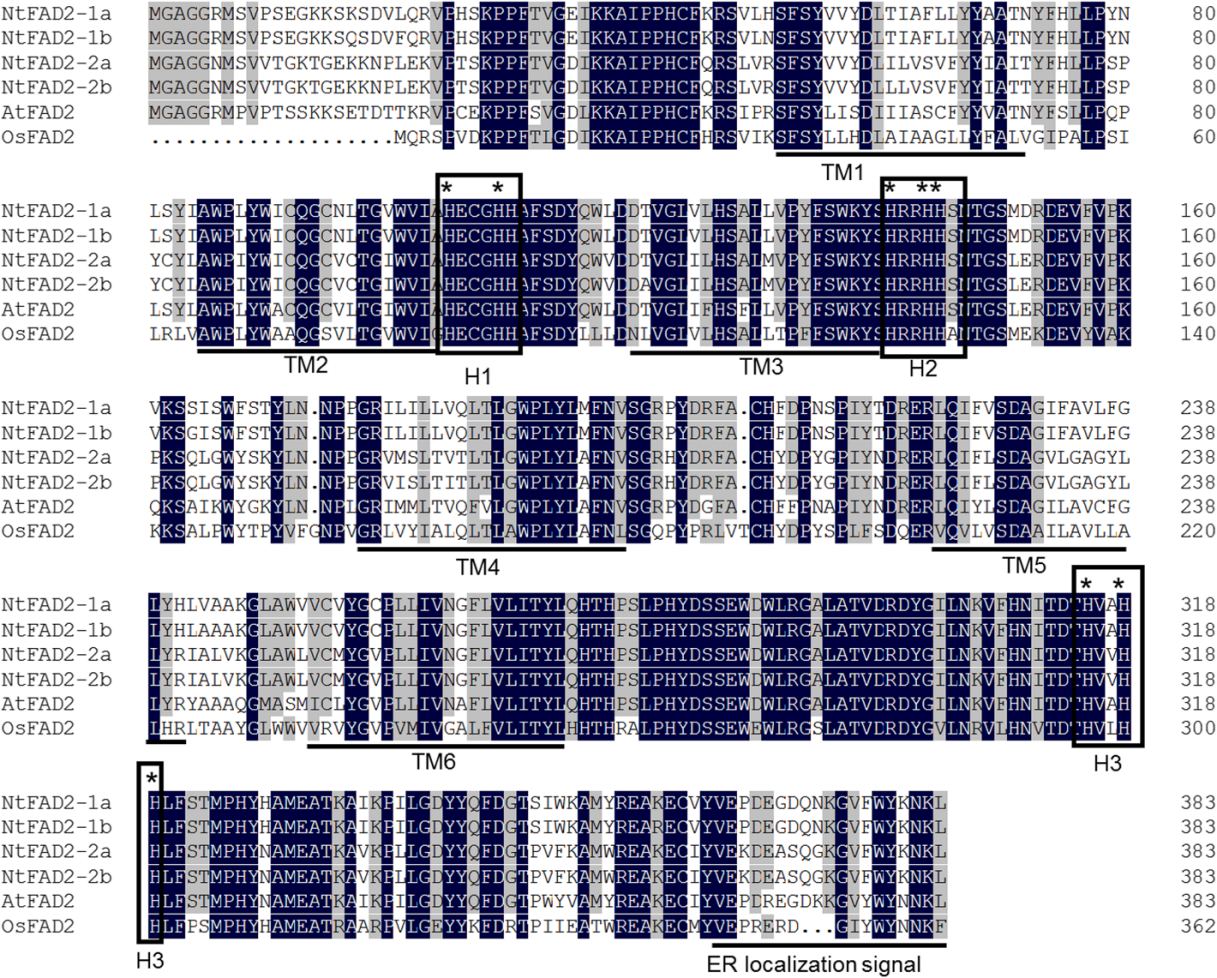
Alignment of NtFAD2–1a, NtFAD2–1b, NtFAD2–2a, NtFAD2–2b, AtFAD2, and OsFAD2 protein sequences. Multiple sequence alignment was performed using DNAMAN 8.0. Black and gray shading indicate conserved and similar amino acids, respectively. Open boxes represent H boxes, while the transmembrane α-helices (TM) and endoplasmic reticulum (ER) localization signal are underlined

Using *Arabidopsis* FAD2 protein (NP_187819) as a query sequence, four FAD2 proteins were identified in tobacco genome. According to the sequence similarity, these four FAD2 protein coding genes were designated as *NtFAD2–1a* (XM_016657141), *NtFAD2–1b* (XM_016639420), *NtFAD2–2a* (NM_001326113), and *NtFAD2–2b* (XM_016659639). Previous studies demonstrated that FAD2 proteins contained six transmembrane domains and eight conserved histidine residues in three H boxes, which were essential for its endoplasmic reticulum (ER) localization and desaturase activity [12, 38]. Six transmembrane domains were predicted in all tobacco FAD2 proteins using TMpred software (https://embnet.vital-it.ch/software/TMPRED_form.html) [39]. In addition, protein sequence alignment showed that the putative transmembrane domains and H boxes of tobacco FAD2 proteins were identical to those regions reported in other FAD2 proteins (Fig. 1). FAD2 protein is an ER membrane-bound fatty acid desaturase, an ER retrieval motif is needed at the C-terminus for ER localization [40, 41]. Sequence analysis showed that tobacco FAD2 proteins contained the conserved ER localization signal (YKNKL) at the C-terminus (Fig. 1). In addition to the high levels of amino acid sequence similarity of tobacco FAD2 proteins, the coding regions also showed a relatively high level sequence identity (~ 85%) (Additional file 1: Figure. S1), which would make functional analysis through RNAi method a challenge.

Tobacco is a natural allotetraploid plant generated by the hybridization of *N. sylvestris* and *N. tomentosiformis*. In order to trace the origin of these four *FAD2* genes, phylogenetic analysis was performed. Our result showed that *NtFAD2–1a* and *NtFAD2–2a* originated from *N. tomentosiformis*, while *NtFAD2–1b* and *NtFAD2–2b* originated from *N. sylvestris* (Additional file 1: Figure. S2).

### NtFAD2–2a and NtFAD2–2b were seed-type FAD2 genes

**Fig. 2:**
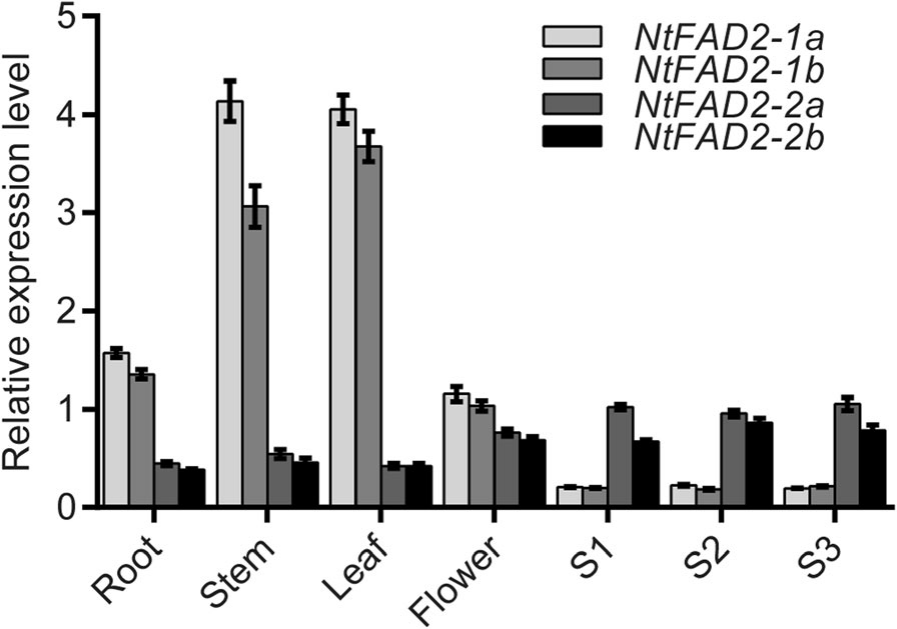
Expression profile of tobacco *FAD2* genes. R, root; L, leaf; S, stem; F, flower; S1, developing seed at 7 days after flowering (DAF); S2, developing seed at 15 DAF; S3, developing seed at 21 DAF. Data are mean ± SD of three biological replicates. *NtGAPDH* gene was amplified as an internal control

To characterize the function of the tobacco *FAD2* genes, real-time qRT-PCR was performed to investigate the expression profiles of *NtFAD2* genes in various organs. Our results showed that *NtFAD2–1a* and *NtFAD2–1b* had a similar expression pattern, *NtFAD2–2a* and *NtFAD2–2b* had a similar expression profile, possibly due to the high levels of sequence identity of the promoter region (Additional file 1: Figure. S3)*. NtFAD2–1a* and *NtFAD2–1b* genes were highly expressed in vegetative tissues and flowers, while the transcript abundance in developing seed was relatively low. *NtFAD2–2a and NtFAD2–2b* genes were detected in all organs, with the highest transcription level in developing seeds (Fig. 2). These results indicated that *NtFAD2–2* genes might be mainly involved in the desaturation of seed storage lipids and *NtFAD2–1* genes might play a more important role in vegetative organs and flowers cell membrane lipid desaturation.

**Fig. 3:**
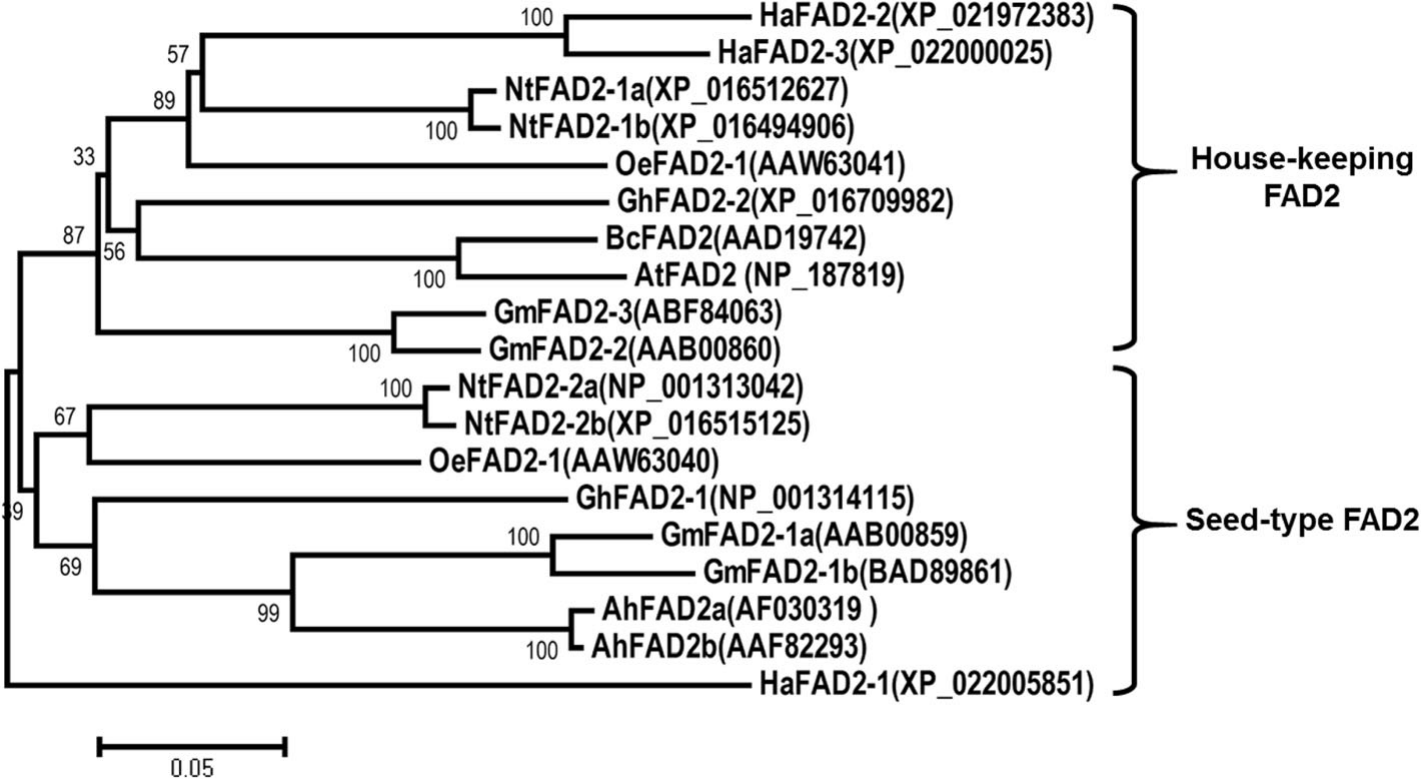
Phylogenetic analysis of FAD2 proteins from various plants. Phylogenetic tree was constructed using the MEGA5.0 software. The letters and numbers listed after FAD2 represent protein accession number. The letters preceding FAD2 indicate the initials of individual species, defined as follows: Ah, *Arachis hypogaea*; At, *Arabidopsis thaliana*; Bc, *Brassica carinata*; Gm, *Glycine max*; Gh, *Gossypium hirsutum*; Ha, *Helianthus annuus*; Oe, *Olea europaea*; Nt, *Nicotiana tabacum*

Previous studies demonstrated that *FAD2* genes could be classified into house-keeping type and seed-type based on phylogenetic analysis. The real time qRT-PCR results indicated that *NtFAD2–2* genes belonged to the seed-type *FAD2* genes. To further confirm this result, phylogenetic tree was constructed based on the protein sequence. Phylogenetic analysis showed that *NtFAD2–1* genes were classified into house-keeping type. The *NtFAD2–2* genes were classified into the same branch with the previously reported seed-type *FAD2* genes from other oil crops, including sunflower, soybean, and cotton (Fig. 3). Taken together, these results demonstrated that tobacco *NtFAD2–2* genes were seed-type *FAD2* genes.

### NtFAD2–2 proteins showed Δ^12^ desaturase activity in yeast cells

**Fig. 4:**
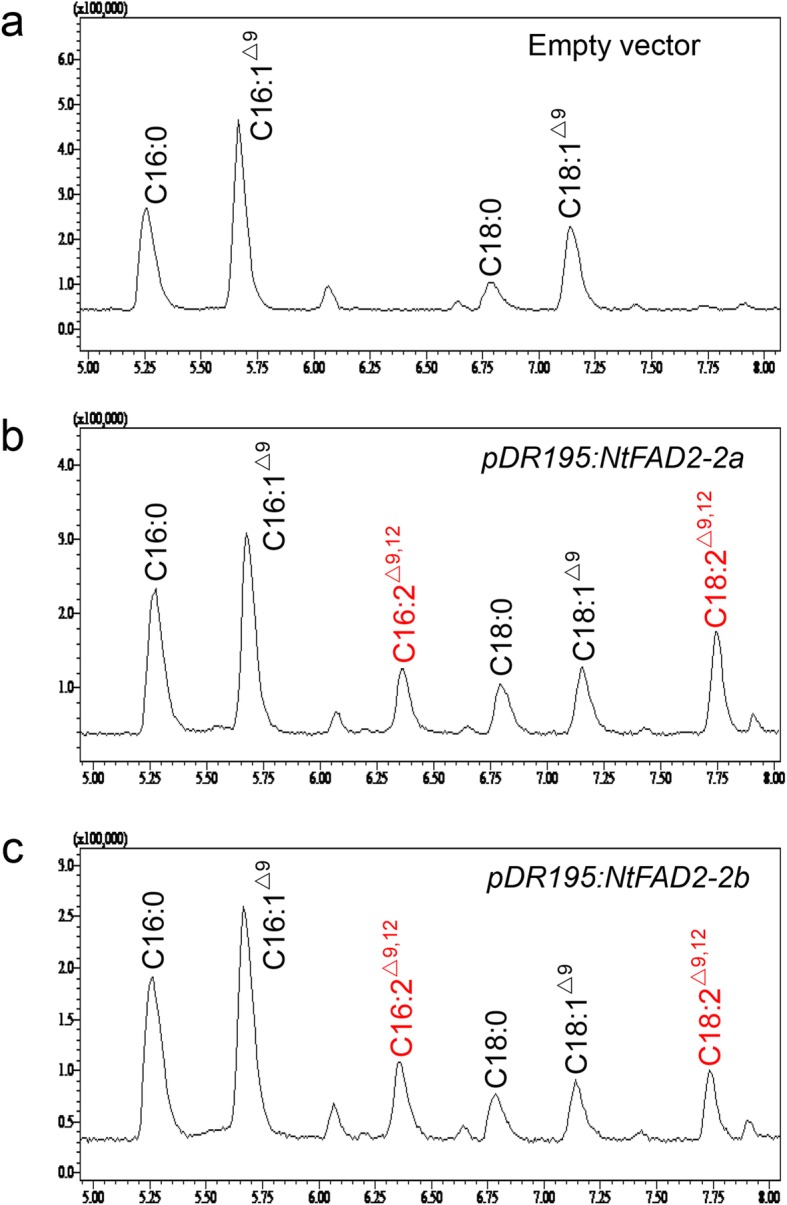
GC-MS analysis of fatty acid methyl esters (FAMEs) from yeast cells expressing *NtFAD2–2a* and *NtFAD2–2b*. **a**, yeast cells transformed with pDR195 empty vector. **b**, yeast cells transformed with pDR195-NtFAD2–2a. **c**, yeast cells transformed with pDR195-NtFAD2–2b

Yeast (*Saccharomyces cerevisiae*) cells had been successfully used for functional characterization of *FAD2* genes from a variety of plant species [16, 17, 42–45]. Due to the lack of *FAD2*-like genes, yeast cells cannot convert oleic acid into linoleic acid. To confirm the desaturation activity of the tobacco seed-type NtFAD2–2 proteins, the coding regions of *NtFAD2–2a* and *NtFAD2–2b* genes were cloned into the yeast expression vector pDR195 and expressed in yeast cells under the control of PMA1 promoter. Yeast cells transformed with the empty vector pDR195 contained only the typical yeast fatty acids, including C16:0, C16:1^Δ9^, C18:0, and C18:1^Δ9^ (Fig. 4a). Expression of both *NtFAD2–2a* and *NtFAD2–2b* genes resulted in two additional peaks in gas chromatograms, corresponding to C16:2^Δ9, 12^ and C18:2^Δ9, 12^ (Fig. 4b, Fig. 4c). In addition, the peak area of C16:1^Δ9^ and C18:1^Δ9^ in the *NtFAD2–2* genes overexpressed yeast cells were noticeably smaller than the corresponding peak in the empty vector transformed cells, which might indicate the conversion of 16:1^Δ9^ and C18:1^Δ9^ into C16:2^Δ9, 12^ and C18:2^Δ9, 12^, respectively (Additional file 2: Table S1). Taken together, these results demonstrated that the seed-type NtFAD2–2a and NtFAD2–2b proteins had Δ^12^ desaturase activity in yeast cells.

### Generation of *NtFAD2–2* knockout mutants using CRISPR-Cas9-mediated genome editing

**Fig. 5:**
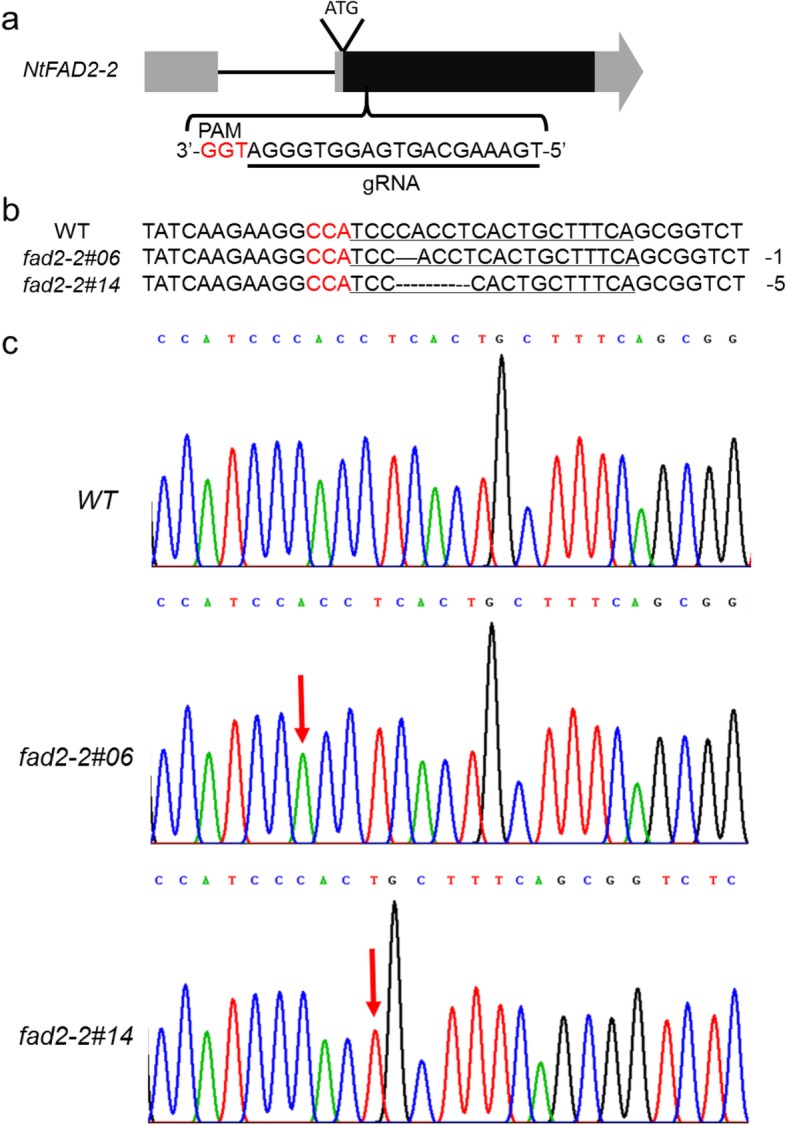
CRISPR-Cas9 mediated *NtFAD2–2 genes* mutation. a, Targeting site and gRNA sequence used for *NtFAD2–2* genes editing. 19 bp gRNA sequence was underlined. The 3 base PAM sequence was in red. **b**, Sequences of wild type and representative mutation types induced at the targeting site of *NtFAD2–2* were presented, respectively. **c**, DNA sequencing peaks of WT and representative mutation types at the targeting site of *NtFAD2–2*, respectively. The red arrows indicate the location of mutations

To further investigate the role of *NtFAD2–2* genes in tobacco seed fatty acid desaturation and to construct a high oleic acid tobacco seed oil material, we generated *NtFAD2–2* knockout lines using CRISPR-Cas9-based genome editing method. Due to the high sequence identity of *NtFAD2–2a* and *NtFAD2–2b* genes, one gRNA targeting the coding sequence of both genes was designed using CRISPR-P 2.0 (Fig. 5a). The CRISPR/Cas9 construct was introduced into tobacco via the Agrobacterium-mediated transformation. Eighteen T0 transgenic lines were obtained by kanamycin selection and two independent homozygous mutated plants for both *NtFAD2–2a* and *NtFAD2–2b* genes (*fad2–2#06* and *fad2–2#14*) were identified through Sanger sequencing. *fad2–2#06* mutant had 1 bp deletion and *fad2–2#14* had 5 bp deletion at 3 bp upstream of the protospacer adjacent motif (PAM) sequence (Fig. 5b, Fig. 5c), which all resulted in a premature stop codon. Using a PCR method, T-DNA construct free plants were selected from 20 T1 segregant plants generated from the self-pollination of each of the two mutant plants, respectively (Additional file 1: Figure. S4). Three T-DNA free homozygous mutant T1 plants from each mutant line were selected for phenotype analysis.

### Oleic acid content was increased in *fad2–2* mutant seeds

**Fig. 6:**
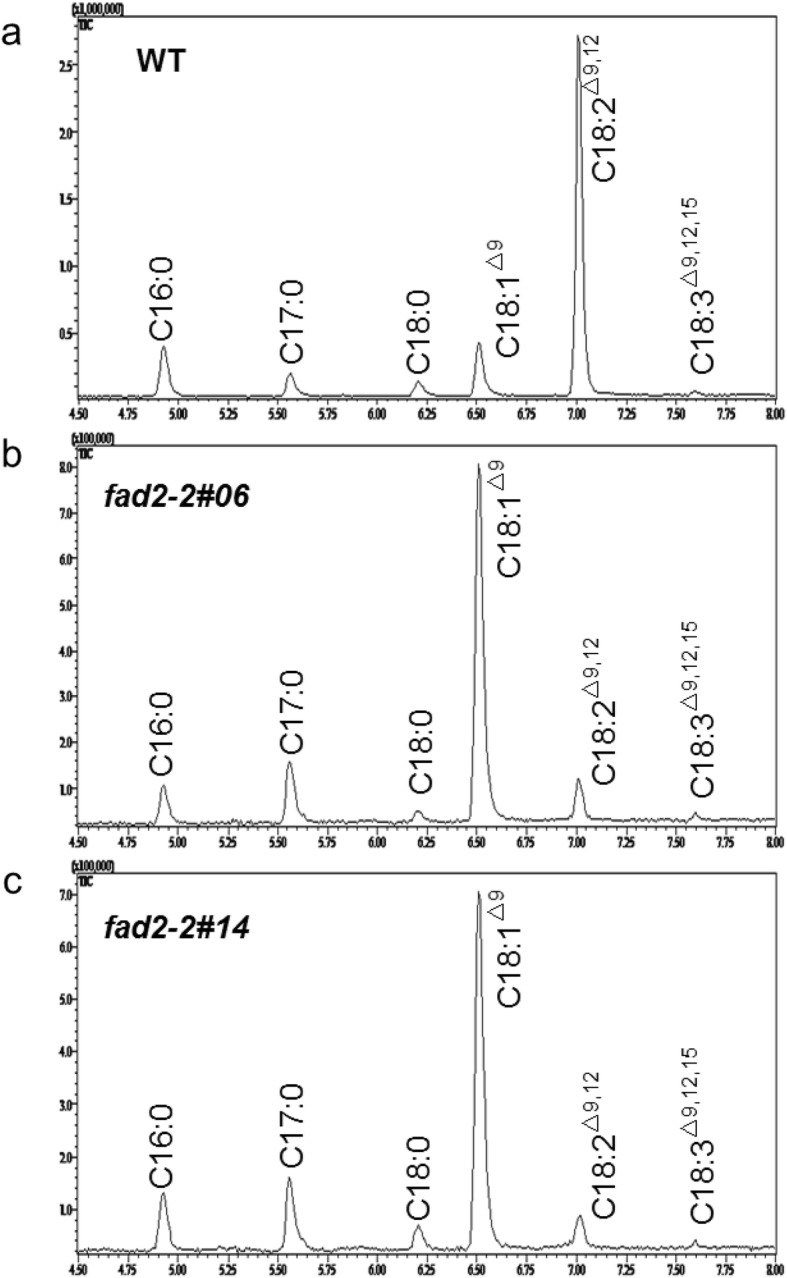
GC-MS analysis of fatty acid methyl esters (FAMEs) from WT and *fad2–2* mutant tobacco seeds. **a**, Fatty acid profile of WT seeds. **b**, Fatty acid profile of *fad2–2#06* mutant seeds. **c**, Fatty acid profile of *fad2–2#14* mutant seeds

**Fig. 7:**
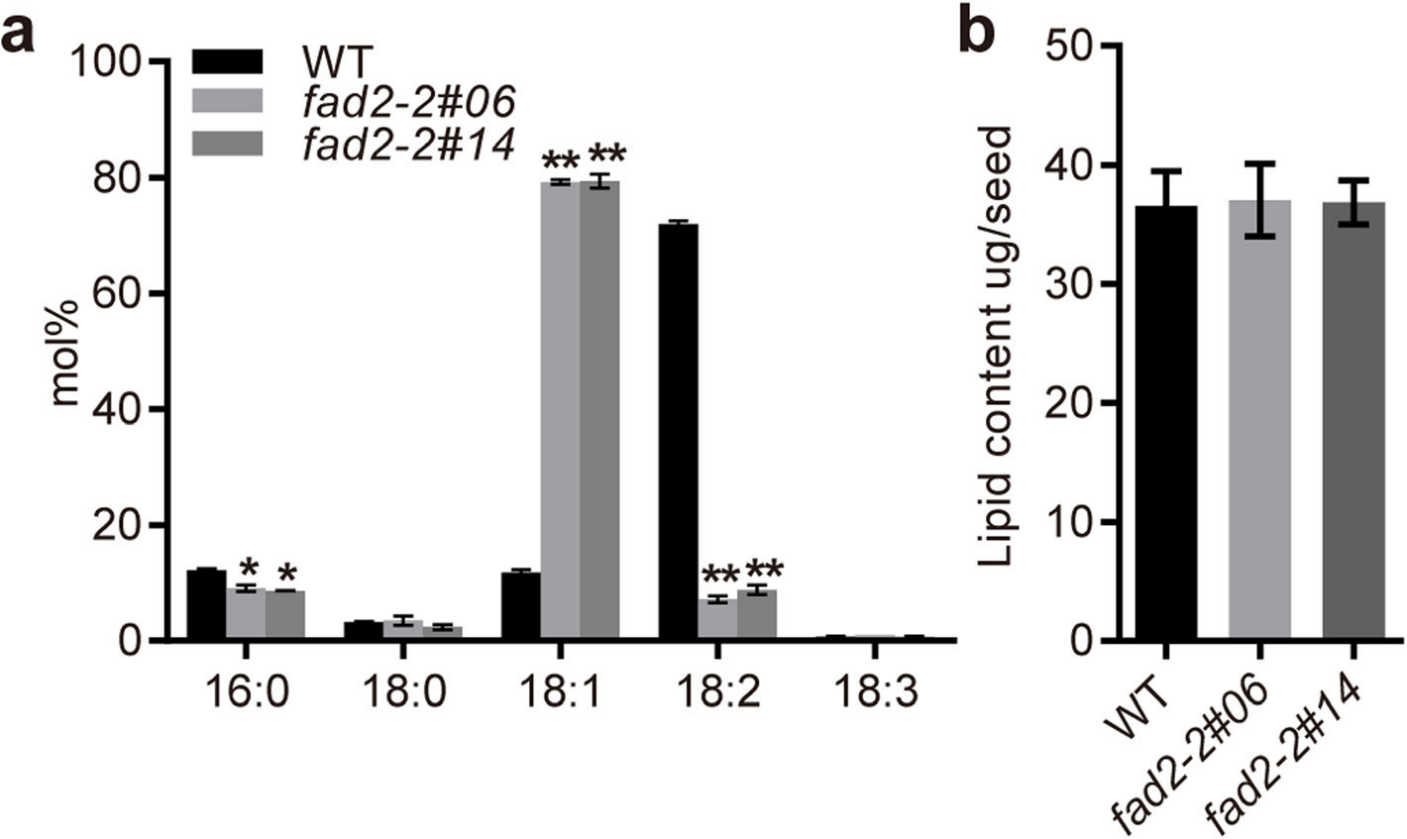
Lipid analysis of WT and *fad2–2* mutant seeds. a, fatty acid composition (mol %) of WT and *fad2–2* mutant seeds. Data are mean ± SD (*n* = 3). Asterisks indicate significant difference compared to the corresponding controls determined by one-way ANOVA with Dunnett’s test (*P < 0.05; **P < 0.01). **b**, Lipid content per seed. Data are mean ± SD (*n* = 3)

Fatty acid profile of mature seeds derived from *fad2–2* mutant plants were analyzed by GC-MS. Compared with the WT plants, seeds derived from both mutant lines showed the expected high oleic acid and low linoleic acid phenotype (Fig. 6). Oleic acid content was increased dramatically from ~ 12% in WT seeds to over 79% in both mutant lines (Fig. 7a). On the contrary, linoleic acid content was significantly reduced from 71.9% in WT plant seeds to 7.1–8.8% in both mutant lines (Fig. 7a). However, linoleic acid was still detected in both mutant lines, the remaining linoleic acid might be generated by the fatty acid desaturase activity provided by the low expression levels of *NtFAD2–1* and plastidial *FAD6* genes in developing seeds. Surprisingly, palmitic acid content was significantly reduced in the *fad2–2* mutant seeds (Fig. 7a). The reduction of palmitic acid level was also observed in *FAD2* suppressed peanut, soybean, and cotton [21, 37, 46], whereas the underlying mechanism of palmitic acid reduction was still unclear.

Previous study showed that the high-oleic acid phenotype of RNAi mediated *FAD2*-silenced tobacco was abated by lower temperature, and the author suggested that the instability of the high-oleic acid phenotype must be partially due to activities of plastidial desaturases [47]. Thus, we examined the fatty acid composition of low temperature treated WT and *fad2–2* mutant seeds, our results showed that the WT seeds showed an increased level of linoleic acid content under low temperature, however, the high-oleic phenotype of *fad2–2* mutant seeds was not affected by low temperature (Table 1).

**Table 1:** Fatty acid composition (mol%) of tobacco seeds from wild type and *fad2–2* mutant plants

	CK			Low temperature treatment		
	WT	*fad2–2#06*	*fad2–2#16*	WT	*fad2–2#06*	*fad2–2#16*
16:0	12.1 ± 0.9	8.6 ± 0.7	8.5 ± 0.6	10.2 ± 0.8	8.3 ± 0.8	8.5 ± 0.9
18:0	3.3 ± 0.5	4.1 ± 0.3	3.7 ± 0.1	3.1 ± 0.4	3.9 ± 0.5	3.4 ± 0.1
18:1^Δ9^	11.6 ± 0.7	79.6 ± 3.6	78.5 ± 2.8	9.1 ± 0.5	78.6 ± 4.2	77.9 ± 3.7
18:2^Δ9, 12^	72.4 ± 3.1	6.8 ± 0.2	8.4 ± 0.6	76.7 ± 4.3	7.2 ± 0.4	9.1 ± 0.8
18:3^Δ9, 12, 15^	0.6 ± 0.06	0.9 ± 0.08	0.8 ± 0.06	0.9 ± 0.08	1.0 ± 0.1	0.9 ± 0.1

16:0, palmitic acid; 18:0, stearic acid; 18:1^Δ9^, oleic acid; 18:2^Δ9, 12^, linoleic acid; 18:2^Δ9, 12, 15^, linolenic acid. The data are the mean ± SD of three independent experiments

### Lipid content was not affected in *fad2–2* mutant seeds

Seed lipid is stored mainly in the form of triacylglycerol (TAG) in oilseed. Due to the inefficient utilization of the engineered fatty acid profile, previous studies showed that total TAG production was usually reduced in multiple genetically modified plant seeds [48–50]. Thus we examined the total lipid content of *fad2–2* mutant seeds. Unexpectedly, the lipid content was not affected in both *fad2–2* mutant lines (Fig. 7b), which indicated that the increased oleic acid could be efficiently incorporated into TAG.

Seed size and seed weight of oil plants were generally positively correlated with seed lipid content [51–53]. We further examined the seed size and weight of WT and *fad2–2* mutant lines. Our results showed that seed size and seed weight were not affected by *NtFAD2–2* genes mutation (Additional file 1: Figure S5). These results were consistent with the seed lipid content.

### Fatty acid profile of leaf was not changed in *fad2–2* mutants

The house-keeping *NtFAD2–1a* and *NtFAD2–1b* genes sequence were highly similar with the seed-type *NtFAD2–2a* and *NtFAD2–2b* genes (Additional file 1: Figure. S1). Probably due to the unspecific property, RNAi mediated *FAD2* genes silencing in tobacco led to increased oleic acid content in leaf fatty acid profile [54], which might affect the stress tolerance of genetically modified plants. In this present paper, we identified *NtFAD2–2a* and *NtFAD2–2b* as seed-type *FAD2* genes and created *fad2–2* mutant through high specific CRISPR-Cas9 system. We suggested that mutation of the seed-type *NtFAD2–2* genes will not affect the fatty acid desaturation in tobacco leaves, because of the function redundancy of *NtFAD2–1* genes in vegetative tissues. In order to confirm this possibility, we examined the leaf fatty acid profile of WT and *fad2–2* mutant lines. The GC-MS results showed that fatty acid composition of leaves from *fad2–2* mutant lines were comparable with that of WT plant (Table 2). Our results indicated that editing the seed-type *FAD2* genes by CRISPR-Cas9 system was a feasible way to engineer the tobacco seed oil without affecting the vegetative tissues.

**Table 2:** fatty acid composition of tobacco leaf from wild type and *fad2–2* mutant plants

Vector	Fatty acid composition (mol%)				
	16:0	18:0	18:1^Δ9^	18:2^Δ9, 12^	18:3^Δ9, 12, 15^
WT	12.6 ± 0.2	4.9 ± 0.1	6.1 ± 0.1	12.2 ± 0.2	64.1 ± 1.7
*fad2–2#06*	12.7 ± 0.3	4.5 ± 0.2	6.3 ± 0.2	12.7 ± 0.1	63.8 ± 1.2
*fad2–2#14*	11.9 ± 0.2	5.1 ± 0.3	5.8 ± 0.1	13.1 ± 0.3	64.8 ± 2.1

16:0, palmitic acid; 18:0, stearic acid; 18:1^Δ9^, oleic acid; 18:2^Δ9, 12^, linoleic acid; 18:2^Δ9, 12, 15^, linolenic acid. Data are means ± SD of three independent experimental replicates

## Discussion

### Tobacco seed-type *FAD2* genes could be used as the target for seed fatty acid profile engineering

Due to the high oil content, tobacco seed was proposed to be a promising feedstock for biodiesel production [2, 4, 9]. However, the high linoleic acid content of tobacco seed oil is problematic from the perspective of oxidative stability, which is important for industrial applications [1, 3]. Low linoleic acid and high oleic acid content are preferred in nearly all oil seed crops breeding, and high oleic acid cultivars have been generated by selecting for or engineering reduced FAD2 activity [55]. In this paper, four *FAD2* genes were firstly identified from tobacco genome (Fig. 1). Both gene expression analysis and phylogenetic analysis showed that *NtFAD2–2a* and *NtFAD2–2b* were seed-type *FAD2* genes (Fig. 2, Fig. 3). Knockout of *NtFAD2–2* genes by CRISPR-Cas9 system led to a high oleic acid and low linoleic acid phenotype in tobacco seed oil (Fig. 6, Fig. 7a). In addition, the leaf fatty acid compositions of *NtFAD2–2* genes edited plant were not affected (Table 2). Our results indicated that seed-type *NtFAD2–2* genes were ideal targets for high oleic acid tobacco seed oil breeding.

### CRISPR-Cas9 system was a useful tool for tobacco breeding

The influence of temperature on the polyunsaturated fatty acid content of storage lipids, which was observed in a number of oilseed crops [56, 57], is considered to be undesirable, since it leads to variation in product quality [58]. Temperature was proposed to affect fatty acid desaturation by regulating the expression of *FAD2* gene [59–62] or influencing the rate of FAD2 protein turnover [63–66]. Previous study showed that the high-oleic acid phenotype of *FAD2*-silenced tobacco was abated at lower temperature [47]. Our results demonstrated that knockout of the tobacco seed-type *NtFAD2–2* genes using the CRISPR-Cas9 system could get a stable and high oleic acid tobacco seed oil (Table 2).

FAD2 catalyzed fatty acid desaturation is a key step in biosynthesis of polyunsaturated fatty acids, including linolenic acid, which is important in maintaining cellular function. Previous studies have demonstrated that *FAD2* genes play crucial roles in response to various abiotic stresses, such as chilling temperature [60, 67–69] and salt [70]. Most recently, *FAD2* genes were demonstrated to be responsible for biotic stress in tomato [55]. The house-keeping *NtFAD2–1* genes sequence were highly similar with the seed-type *NtFAD2–2* genes. Probably due to the low specificity of RNAi-mediated gene silencing method, the fatty acid composition of transgenic tobacco leaf was also altered, which might affect the stress tolerance of transgenic lines [47, 54]. CRISPR-Cas9 system mediated gene knockout is more specific, our results showed that fatty acid profile of leaves from mutant lines were not affected (Table 2). Thus, the high oleic acid tobacco mutant lines generated by CRISPR-Cas9 system had no side effects and could be used as basal material for further lipid engineering.

### Tobacco *fad2–2* mutant lines could be further engineered to accumulate unusual fatty acid

FAD2 protein adds a double bond at the Δ^12^ position of oleic acid. Plants accumulating unusual fatty acids possess divergent forms of the Δ^12^-desaturase (FAD2) that are able to catalyze alternative modifications at the Δ^12^ position of oleic acid, such as the formation of a carbon-carbon triple bond or a conjugated double bond arrangement, or the insertion of an epoxy bridge or hydroxyl group. Due to the unique physical and chemical properties, unusual fatty acids could be used as additives or raw materials for numerous industrial products, such as lubricants, plastics, and inks [71]. However, unusual fatty acids are mainly produced in plants which has not been domesticated as crops, such as castor bean and tung tree. In order to meet the increasingly need, recent research efforts have been focused on engineering desirable unusual fatty acids using biotechnology in existing oilseeds [71–73]. *sn*-2- 18:1-PC is used as substrate for both FAD2 and divergent forms of FAD2 proteins. In order to maximum the production of unusual fatty acids, the endogenous FAD2 activity has to be abolished. In this paper, high-oleic acid tobacco seed oil mutants was generated by CRISPR-Cas9 system, and these mutant lines could be used for unusual fatty aicds engineering.

## Conclusion

In summary, four *FAD2* genes were identified in tobacco genome for the first time, and *NtFAD2–2a* and *NtFAD2–2b* genes were confirmed to be seed-type *FAD2* genes. Compared with the RNAi technology, two stable and high-oleic acid tobacco seed oil lines were generated using CRISPR-Cas9-mediated gene knockout method. In addition, the leaf fatty acid profile of the mutants created by CRISPR-Cas9 system was not affected. The mutants generated in this present study could be used for advanced engineering to produce unusual fatty acid.

## Methods

### Plant materials and growth condition

Wild type (WT) tobacco (*Nicotiana tabacum* L. cv. K326) was provided by the Yunnan Academy of Tobacco Agriculture Science (http://ynycnky.yn-tobacco.com/). Dr. Changjun Huang from Key Laboratory of Tobacco Biotechnological Breeding in Yunnan Academy of Tobacco Agriculture Science undertook the formal identification of the samples and provided details of specimen deposited. WT tobacco was used for genetic transformation. WT and transgenic or mutant tobacco plants were grown in the greenhouse at 25 °C with 16 h light and 8 h dark. For low temperature treatment, developing seeds at 20 days after pollination were collected and cultivated on 1/2 MS medium supplemented with 2% sucrose at 18 °C for 24 h in the dark. The control treatment temperature was 25 °C.

### Isolation and sequence analysis of *FAD2* genes in tobacco

To identify the putative *FAD2* genes in tobacco, *Arabidopsis* FAD2 protein (accession number: NP_187819) was used as a query sequence to blast against the tobacco genome database (https://solgenomics.net/organism/Nicotiana_tabacum/genome) under the default parameters. Homologous protein sequences with an identity score higher than 70% were chosen for further analysis. The target sequences were further confirmed through BLAST program at National Center for Biotechnology Information (NCBI). Sequence alignment was performed with DNAMAN software. Phylogenetic tree was constructed with the neighbor-joining algorithm using the MEGA5.0 [74]. Bootstrap analysis with 1000 replicates was performed to test the significance of nodes.

### RNA isolation and quantitative RT-PCR (qRT-PCR)

Plant tissues were sampled and ground immediately in liquid N_2_, total RNA was extracted using the Plant Total RNA Isolation Kit (Foregene, China) and reverse-transcribed into cDNA with PrimeScript™ RT reagent Kit (Takara, Dalian). qRT-PCR program was performed using SYBR Premix Ex Taq™ II (Tli RNaseH Plus) (Takara, Dalian) on a CFX96 real-time PCR system (Bio-Rad, USA). The qRT-PCR cycling began with one cycle at 95 °C for 2 min, followed by 40 cycles at 95 °C for 30 s, 55 °C for 30 s, 72 °C for 30 s. The house-keeping gene, *NtGAPDH* (XM_016643257), was used as the internal control. Three biological and three technical replicates were performed. All primer used for qRT-PCR were listed in Additional file 2: Table S2.

### Expression of *NtFAD2–2* genes in *S. cerevisiae*

The coding regions of *NtFAD2–2a* and *NtFAD2–2b* genes were amplified and inserted into pDR195 vector. The reconstructed vectors were named as pDR195:NtFAD2–2a and pDR195:NtFAD2–2b, respectively. The BY4741 strain of *Saccharomyces cerevisiae* was transformed with these vectors using the lithium acetate method and selected on minimal agar plates lacking uracil [75]. Isolated colonies were inoculated in 10 ml complete minimal drop out-Uracil medium containing 2% glucose. When the culture reached at stationary phase, yeast cells were harvested by centrifugation at 1500 g for 5 min at 4 °C, and washed once with distilled water. Primers used for vectors construction were listed in Additional file 2: Table S2.

### Vector construction

For CRISPR-Cas9 vector construction, guide RNA (gRNA) targeting the coding region of *NtFAD2–2* genes was designed using a CRISPR-P 2.0 (http://crispr.hzau.edu.cn/CRISPR2/) [76]. gRNA was inserted into the pKSE401 vector using Golden-gate assembly method as described previously [77]. The reconstructed vector (named as *pKSE401-NtFAD2–2*) was confirmed using the Sanger sequencing and introduced into *Agrobacterium tumefaciens* strain GV3101. Primers used for vectors construction were listed in Additional file 2: Table S2.

### Tobacco genetic transformation and selection

Transgenic tobacco was generated by the Agrobacterium-mediated transformation as previously reported [78]. T0 generation lines were selected on media with 50 mg/L kanamycin. Mutation selection was performed as previous reported with some modification [79]. Briefly, genomic DNA was extracted from the leaves of the kanamycin-resistant T0 transgenic plants using the Plant DNA Isolation Kit (Foregene, China). To select mutant transgenic plants, PCR amplification was performed using a primer pair flanking the gRNA target site of *NtFAD2–2* loci (the forward primer annealing at 39 bp downstream of the start codon, and the reverse primer annealing at 441 bp downstream of the start codon), and the generated PCR products were directly sequenced using the Sanger method. Mutant plants were selected by comparing the amplicon sequences and the WT template. Twenty T1 segregant plants generated from the self-pollination of T0 mutated plants were screened for the presence/absence of the T-DNA using PCR assay directed to the *Cas9* gene sequence. Three T-DNA free homozygous mutant T1 plants were selected for phenotype analysis. The PCR primers used in these steps are listed in Additional file 2: Table S2.

### Lipid analysis

To analyze lipids from yeast cells, tobacco leaves, and tobacco seeds, fatty acid methyl esters (FAMEs) were prepared as previously described [80], and then quantitatively analyzed by gas chromatography mass spectrometry (GC-MS) (GC-2010, DAOJING). The GC conditions were as follows: split injection (1: 20), injector temperature 220 °C, oven temperature program 150 °C for 1 min, then increasing by 10 °C min^− 1^ to 200 °C, holding at 200 °C for 1 min, then increasing by 5 °C min^− 1^ to 210 °C, held for 1 min.

### Seed weight and size measurement

Mature seeds were harvested from WT and mutant tobacco plants grown under the same conditions. Fifty seeds per group were weighed carefully on analytical balance, per seed weight was calculated by dividing the seed number. Values were given as mean ± SD. Mature seeds were photographed with a stereoscopic microscope (Leica, Germany) and Image J software was used to measure the seed size. Values were given as mean ± SD.

## Supplementary information

**Additional file 1 Figure S1.** Protein coding sequence alignment of *NtFAD2–1a*, *NtFAD2–1b*, *NtFAD2–2a*, and *NtFAD2–2b*. **Figure S2.** Phylogenetic analysis of FAD2 proteins from *N. tabacum*, *N. tomentosiformis, N. sylvestris, and S. lycopersicum*. **Figure S3.** Alignment of promoter sequence. **a**, Alignment of promoter sequence of *NtFAD2–1a* and *NtFAD2–1b*. **b**, Alignment of promoter sequence of *NtFAD2–2a* and *NtFAD2–2b*. **Figure S4.** PCR-based identification of T-DNA-free T1 segregants using primers directed at the Kan sequence. Segregants among (a) *fad2–2#06* plants, and (b) *fad2–2#16* plants. The vector *pKSE401-FAD2–2* was used as the positive control (CK+) and DNA from WT plant as the negative control (CK-). M: DNA ladder. **Figure S5.** Phenotype of WT and *fad2–2* mutant tobacco seed. **a**, Mature seeds from WT and *fad2–2* mutant lines. Bar = 500 μm. **b**, Average seed length. Data are mean ± SD (*n* = 30). **c**, Average seed width. Data are mean ± SD (*n* = 30). **d**, Average seed weight. Values are mean ± SD of five individual measurements of 50 seeds/replicate.

**Additional file 2 Table S1.** Fatty acid profile of yeast cells transformed with *NtFAD2–2* genes. **Table S2.** List of primers used in this study.

## Data Availability

All data generated or analysed during this study are included in this published article (and its supplementary information files). The datasets used and/or analysed during the current study available from the corresponding author on reasonable request.

## Abbreviations

CRISPR: Clustered regularly interspaced short palindromic repeats
DAF: Days after flowering
ER: Endoplasmic reticulum
FAD: Fatty acid desaturase
FAMEs: Fatty acid methyl esters
GC-MS: Gas chromatography-mass spectrometry
NCBI: National center for biotechnology information
PC: Phosphatidylcholine
RNAi: RNA interference
SD: Standard deviation
TAG: Triacylglycerol
T-DNA: Transfer-DNA

## References

[1] Giannelos PN, Zannikos F, Stournas S, Lois E, Anastopoulos G (2002). Tobacco seed oil as an alternative diesel fuel: physical and chemical properties. Industrial Crops Products.

[2] Veljkovic V, Lakicevic S, Stamenkovic O, Todorovic Z, Lazic M (2006). Biodiesel production from tobacco (*Nicotiana tabacum* L.) seed oil with a high content of free fatty acids. Fuel..

[3] Kumar M, Sharma MP (2016). Selection of potential oils for biodiesel production. Renewable Sust Energy Rev.

[4] Banković-Ilić IB, Stamenković OS, Veljković VB (2012). Biodiesel production from non-edible plant oils. Renew Sust Energ Rev.

[5] Usta N, Aydoğan B, Çon AH, Uğuzdoğan E, Özkal SG (2011). Properties and quality verification of biodiesel produced from tobacco seed oil. Energy Convers Manag.

[6] Zlatanov M, Angelova M, Antova G (2007). Lipid composition of tobacco seeds. Bulgarian J Agric Sci.

[7] Azam M, Habib U, Hamid M (2010). Fatty acid composition of tobacco seed oil and synthesis of alkyd resin. Chin J Chem.

[8] Chu H, Tso TC (1968). Fatty acid composition in tobacco I. Green tobacco plants. Plant Physiol.

[9] Grisan S, Polizzotto R, Raiola P, Cristiani S, Ventura F, di Lucia F, Zuin M, Tommasini S, Morbidelli R, Damiani F (2016). Alternative use of tobacco as a sustainable crop for seed oil, biofuel, and biomass. Agron Sustain Dev.

[10] Carvalho FS, Fornasier F, Leitão JOM, Moraes JAR, Schneider RCS (2019). Life cycle assessment of biodiesel production from solaris seed tobacco. J Clean Prod.

[11] Durrett TP, Benning C, Ohlrogge J (2008). Plant triacylglycerols as feedstocks for the production of biofuels. Plant J.

[12] Okuley J, Lightner J, Feldmann K, Yadav N, Lark E, Browse J (1994). Arabidopsis FAD2 gene encodes the enzyme that is essential for polyunsaturated lipid synthesis. Plant Cell.

[13] Li L, Wang X, Gai J, Yu D (2007). Molecular cloning and characterization of a novel microsomal oleate desaturase gene from soybean. J Plant Physiol.

[14] Lee KR, Kim SH, Go YS, Jung SM, Roh KH, Kim JB, Suh MC, Lee S, Kim HU (2012). Molecular cloning and functional analysis of two *FAD2* genes from American grape (*Vitis labrusca* L.). Gene..

[15] Yang Q, Fan C, Guo Z, Qin J, Wu J, Li Q, Fu T, Zhou Y (2012). Identification of FAD2 and FAD3 genes in *Brassica napus* genome and development of allele-specific markers for high oleic and low linolenic acid contents. Theor Appl Genet.

[16] Rodríguez-Rodríguez MF, Salas JJ, Venegas-Calerón M, Garcés R, Martínez-Force E (2016). Molecular cloning and characterization of the genes encoding a microsomal oleate Δ12 desaturase (CsFAD2) and linoleate Δ15 desaturase (CsFAD3) from *Camelina sativa*. Ind Crop Prod.

[17] Wu P, Zhang S, Zhang L, Chen Y, Li M, Jiang H, Wu G (2013). Functional characterization of two microsomal fatty acid desaturases from *Jatropha curcas* L. J Plant Physiol.

[18] Aadi Moolam R, Singh A, Shelke RG, Scott PT, Gresshoff PM, Rangan L (2016). Identification of two genes encoding microsomal oleate desaturases (FAD2) from the biodiesel plant *Pongamia pinnata* L. Trees..

[19] Cahoon EB, Carlson TJ, Ripp KG, Schweiger BJ, Cook GA, Hall SE, Kinney AJ (1999). Biosynthetic origin of conjugated double bonds: production of fatty acid components of high-value drying oils in transgenic soybean embryos. PNAS..

[20] Hee JJ, Hyojin K, Young Sam G, Saet Buyl L, Cheol-Goo H, Hyun Uk K, Chung SM (2011). Identification of functional *BrFAD2-1* gene encoding microsomal delta-12 fatty acid desaturase from *Brassica rapa* and development of *Brassica napus* containing high oleic acid contents. Plant Cell Rep.

[21] Liu Q, Singh SP, Green AG (2002). High-stearic and high-oleic cottonseed oils produced by hairpin RNA-mediated post-transcriptional gene silencing. Plant Physiol.

[22] Yin D, Deng S, Zhan K, Cui D (2007). High-oleic peanut oils produced by hpRNA-mediated gene silencing of oleate desaturase. Plant Mol Biol Report.

[23] Srinivas B, James Robertson P, Pushkar S, Surinder PS (2012). Modification of seed oil composition in *Arabidopsis* by artificial microRNA-mediated gene silencing. Front Plant Sci.

[24] Haun W, Coffman A, Clasen BM, Demorest ZL, Lowy A, Ray E, Retterath A, Stoddard T, Juillerat A, Cedrone F (2015). Improved soybean oil quality by targeted mutagenesis of the fatty acid desaturase 2 gene family. Plant Biotechnol J.

[25] Lee YH, Park W, Kim KS, Jang YS, Lee JE, Cha YL, Moon YH, Song YS, Lee K (2018). EMS-induced mutation of an endoplasmic reticulum oleate desaturase gene (*FAD2–2*) results in elevated oleic acid content in rapeseed (*Brassica napus* L.). Euphytica.

[26] Wells R, Trick M, Soumpourou E, Clissold L, Morgan C, Werner P, Gibbard C, Clarke M, Jennaway R, Bancroft I (2014). The control of seed oil polyunsaturate content in the polyploid crop species *Brassica napus*. Mol Breed.

[27] Dinushika T, Scott D, Evelyn L, Gordon R, Helen B, You FM, Sylvie C (2013). Genetic variation of six desaturase genes in flax and their impact on fatty acid composition. Theoretical Appl Genet.

[28] Anh-Tung P (2012). J Grover S, Bilyeu KD. Combinations of mutant *FAD2* and *FAD3* genes to produce high oleic acid and low linolenic acid soybean oil. Theoretical Appl Genet.

[29] Okuzaki A, Ogawa T, Koizuka C, Kaneko K, Inaba M, Imamura J, Koizuka N (2018). CRISPR/Cas9-mediated genome editing of the fatty acid desaturase 2 gene in *Brassica napus*. Plant Physiol Biochem.

[30] Cong L, Ran FA, Cox D, Lin S, Barretto R, Habib N, Hsu PD, Wu X, Jiang W, Marraffini LA (2013). Multiplex genome engineering using CRISPR/Cas systems. Science..

[31] Jaganathan D, Ramasamy K, Sellamuthu G, Jayabalan S, Venkataraman G (2018). CRISPR for crop improvement: an update review. Front Plant Sci.

[32] Ma X, Zhu Q, Chen Y, Liu YG (2016). CRISPR/Cas9 platforms for genome editing in plants: developments and applications. Mol Plant.

[33] Boettcher M, McManus MT (2015). Choosing the right tool for the job: RNAi, TALEN, or CRISPR. Mol Cell.

[34] Thomas G, Gersbach CA, Barbas CF (2013). ZFN, TALEN, and CRISPR/Cas-based methods for genome engineering. Trends Biotechnol.

[35] Jiang WZ, Henry IM, Lynagh PG, Comai L, Cahoon EB, Weeks DP (2017). Significant enhancement of fatty acid composition in seeds of the allohexaploid, *Camelina sativa*, using CRISPR/Cas9 gene editing. Plant Biotechnol J.

[36] Yuan M, Zhu J, Gong L, He L, Lee C, Han S, Chen C, He G (2019). Mutagenesis of *FAD2* genes in peanut with CRISPR/Cas9 based gene editing. BMC Biotechnol.

[37] Do PT, Nguyen CX, Bui HT, Tran LTN, Stacey G, Gillman JD, Zhang ZJ, Stacey MG (2019). Demonstration of highly efficient dual gRNA CRISPR/Cas9 editing of the homeologous *GmFAD2-1A* and *GmFAD2-1B* genes to yield a high oleic, low linoleic and alpha-linolenic acid phenotype in soybean. BMC Plant Biol.

[38] Shanklin J, Whittle E, Fox BG (1994). Eight histidine residues are catalytically essential in a membrane-associated iron enzyme, stearoyl-CoA desaturase, and are conserved in alkane hydroxylase and xylene monooxygenase. Biochemistry..

[39] Hofman K, Stoffel W (1993). TMBASE-A database of membrane spanning protein segments. Biol Chem Hoppe Seyler.

[40] McCartney AW, Dyer JM, Dhanoa PK, Kim PK, Andrews DW, McNew JA, Mullen RT (2004). Membrane-bound fatty acid desaturases are inserted co-translationally into the ER and contain different ER retrieval motifs at their carboxy termini. Plant J.

[41] Nayeri FD, Yarizade K (2014). Bioinformatics study of delta-12 fatty acid desaturase 2 (FAD2) gene in oilseeds. Mol Biol Rep.

[42] Cao S, Zhou XR, Wood CC, Green AG, Singh SP, Liu L, Liu Q (2013). A large and functionally diverse family of *FAD2* genes in safflower (*Carthamus tinctorius* L.). BMC Plant Biology.

[43] Pirtle IL, Kongcharoensuntorn W, Nampaisansuk M, Knesek JE, Chapman KD, Pirtle RM (2001). Molecular cloning and functional expression of the gene for a cotton Δ-12 fatty acid desaturase (FAD2). Biochim Biophys Acta.

[44] Xue Y, Yin N, Chen B, Liao F, Win AN, Jiang J, Wang R, Jin X, Lin N, Chai Y (2017). Molecular cloning and expression analysis of two *FAD2* genes from chia (*Salvia hispanica*). Acta Physiol Plant.

[45] Xue Y, Zhang X, Wang R, Chen B, Jiang J, Win AN, Chai Y (2017). Cloning and expression of *Perilla frutescens FAD2* gene and polymorphism analysis among cultivars. Acta Physiol Plant.

[46] Wang ML, Barkley NA, Chen Z, Pittman RN (2011). *FAD2* gene mutations significantly alter fatty acid profiles in cultivated peanuts (*Arachis hypogaea*). Biochem Genet.

[47] Zhang L, Lu H, Liu C, Xue F, Yang J, Ma L, Yang M (2016). Lipid desaturation in prokaryotic pathway abates the high-oleic phenotype of *FAD2*-silenced tobacco at lower temperature. J Plant Biochem Biotechnol.

[48] Dauk M, Lam P, Kunst L, Smith MA (2007). A *FAD2* homologue from *Lesquerella lindheimeri* has predominantly fatty acid hydroxylase activity. Plant Sci.

[49] Bates PD, Johnson SR, Xia C, Jia L, Jeong-Won N, Jaworski JG, Ohlrogge JB, John B (2014). Fatty acid synthesis is inhibited by inefficient utilization of unusual fatty acids for glycerolipid assembly. PNAS..

[50] Li R, Yu K, Wu Y, Tateno M, Hatanaka T, Hildebrand DF (2012). *Vernonia* DGATs can complement the disrupted oil and protein metabolism in epoxygenase-expressing soybean seeds. Metab Eng.

[51] Lee E-J, Oh M, Hwang J-U, Li-Beisson Y, Nishida I, Lee Y (2017). Seed-specific overexpression of the pyruvate transporter *BASS2* increases oil content in *Arabidopsis* seeds. Front Plant Sci.

[52] Jako C, Kumar A, Wei Y, Zou J, Barton DL, Giblin EM, Covello PS, Taylor DC (2001). Seed-specific over-expression of an *Arabidopsis* cDNA encoding a diacylglycerol acyltransferase enhances seed oil content and seed weight. Plant Physiol.

[53] Kim S, Yamaoka Y, Ono H, Kim H, Shim D, Maeshima M, Martinoia E, Cahoon EB, Nishida I, Lee Y (2013). AtABCA9 transporter supplies fatty acids for lipid synthesis to the endoplasmic reticulum. PNAS..

[54] Yang M, Zheng G, Zhang F, Xu Y (2006). *FAD2*-silencing has pleiotropic effect on polar lipids of leaves and varied effect in different organs of transgenic tobacco. Plant Sci.

[55] Lee MW, Padilla CS, Gupta C, Galla A, Pereira A, Li J, Goggin FL (2020). The *FATTY ACID DESATURASE2* family in tomato contributes to primary metabolism and stress responses. Plant Physiol.

[56] Canvin DT (2011). The effect of temperature on the oil content and fatty acid composition of the oils from several oil seed crops. Can J Bot.

[57] Carver BF, Burton JW, Carter TE, Wilson RF (1986). Response to environmental variation of soybean lines selected for altered unsaturated fatty acid composition. Crop Sci.

[58] Singer SD, Zou J, Weselake RJ (2016). Abiotic factors influence plant storage lipid accumulation and composition. Plant Sci.

[59] Martz F, Kiviniemi S, Palva TE, Sutinen ML (2006). Contribution of omega-3 fatty acid desaturase and 3-ketoacyl-ACP synthase II (KASII) genes in the modulation of glycerolipid fatty acid composition during cold acclimation in birch leaves. J Exp Bot.

[60] Kargiotidou AD (2008). Dimitra, Galanopoulou D, Tsaftaris a, Farmaki T. low temperature and light regulate *delta 12* fatty acid desaturases (FAD2) at a transcriptional level in cotton (*Gossypium hirsutum*). J Exp Bot.

[61] Román Á, Andreu V, Hernández ML, Lagunas B, Picorel R, Martínezrivas JM, Alfonso M (2012). Contribution of the different omega-3 fatty acid desaturase genes to the cold response in soybean. J Exp Bot.

[62] Zhu Y, Cao Z, Fei X, Yi H, Chen M, Guo W, Zhou W, Zhu J, Meng J, Zou J (2012). Analysis of gene expression profiles of two near-isogenic lines differing at a QTL region affecting oil content at high temperatures during seed maturation in oilseed rape ( *Brassica napus* L.). Theoretical Appl Genet.

[63] Dyer JM, Chapital DC, Cary JW, Pepperman AB (2001). Chilling-sensitive, post-transcriptional regulation of a plant fatty acid desaturase expressed in yeast. Biochem Biophys Res Commun.

[64] Martinez-Rivas JM, Sanchez-Garcia ASicardo MD, Garcia-Diaz MT (2010). Oxygen-independent temperature regulation of the microsomal oleate desaturase (FAD2) activity in developing sunflower (*Helianthus annuus*) seeds. Physiol Plant.

[65] Tang G, Novitzky W, Griffin H, Huber S, Dewey R (2010). Oleate desaturase enzymes of soybean: evidence of regulation through differential stability and phosphorylation. Plant J.

[66] O'Quin JB, Linda B, Daiyuan Z, Shockey JM, Gidda SK, Spencer F, Chapman KD, Mullen RT, Dyer JM (2011). Temperature-sensitive post-translational regulation of plant omega-3 fatty-acid desaturases is mediated by the endoplasmic reticulum-associated degradation pathway. J Biol Chem.

[67] Miquel MF, Browse JA (1994). High-oleate oilseeds dail to develop at low temperature. Plant Physiol.

[68] Shi J, Cao Y, Fan X, Min L, Wang Y, Feng M (2012). A rice microsomal delta-12 fatty acid desaturase can enhance resistance to cold stress in yeast and *Oryza sativa*. Mol Breed.

[69] Zhang YM, Wang CC, Hu HH, Yang L (2011). Cloning and expression of three fatty acid desaturase genes from cold-sensitive lima bean (*Phaseolus lunatus* L.). Biotechnol Lett.

[70] Zhang J, Liu H, Sun J, Li B, Zhu Q, Chen S, Zhang H (2012). *Arabidopsis* fatty acid desaturase FAD2 is required for salt tolerance during seed germination and early seedling growth. PLoS One.

[71] Lee KR, Chen GQ, Kim HU (2015). Current progress towards the metabolic engineering of plant seed oil for hydroxy fatty acids production. Plant Cell Rep.

[72] Aznar-Moreno JA, Durrett TP (2017). Review: metabolic engineering of unusual lipids in the synthetic biology era. Plant Sci.

[73] Snapp AR, Lu C (2012). Engineering industrial fatty acids in oilseeds. Front Biol.

[74] Tamura K, Peterson D, Peterson N, Stecher G, Nei M, Kumar S (2011). MEGA5: molecular evolutionary genetics analysis using maximum likelihood, evolutionary distance, and maximum parsimony methods. Mol Biol Evol.

[75] Elble R (1992). A simple and efficient procedure for transformation of yeasts. Biotechniques..

[76] Liu H, Ding Y, Zhou Y, Jin W, Xie K, Chen LL (2017). CRISPR-P 2.0: an improved CRISPR-Cas9 tool for genome editing in plants. Mol Plant.

[77] Xing HL, Dong L, Wang ZP, Zhang HY, Han CY, Liu B, Wang XC, Chen QJ (2014). A CRISPR/Cas9 toolkit for multiplex genome editing in plants. BMC Plant Biol.

[78] Horsch RB (1985). A simple and general method for transferring genes into plants. Science..

[79] Barman HN, Sheng Z, Fiaz S, Zhong M, Wu Y, Cai Y, Wang W, Jiao G, Tang S, Wei X (2019). Generation of a new thermo-sensitive genic male sterile rice line by targeted mutagenesis of *TMS5* gene through CRISPR/Cas9 system. BMC Plant Biol.

[80] Li Y, Beisson F, Pollard M, Ohlrogge J (2006). Oil content of *Arabidopsis* seeds: the influence of seed anatomy, light and plant-to-plant variation. Phytochemistry..

