# Supplementary material for: Design of high-oleic tobacco (Nicotiana tabacum L.) seed oil by CRISPR-Cas9-mediated knockout of NtFAD2–2
Source: BMC Plant Biol. 2020 May 25;20:233. doi: 10.1186/s12870-020-02441-0 (PMC7249356; doi:10.1186/s12870-020-02441-0)
Supplement: Supplementary file 1 — Additional file 1 Figure S1. Protein coding sequence alignment of NtFAD2–1a, NtFAD2–1b, NtFAD2–2a, and NtFAD2–2b. Figure S2. Phylogenetic analysis of FAD2 proteins from N. tabacum, N. tomentosiformis, N. sylvestris, and S. lycopersicum. Figure S3. Alignment of promoter sequence. a, Alignment of promoter sequence of NtFAD2–1a and NtFAD2–1b. b, Alignment of promoter sequence of NtFAD2–2a and NtFAD2–2b. Figure S4. PCR-based identification of T-DNA-free T1 segregants using primers directed at the Kan sequence. Segregants among (a) fad2–2#06 plants, and (b) fad2–2#16 plants. The vector pKSE401-FAD2–2 was used as the positive control (CK+) and DNA from WT plant as the negative control (CK-). M: DNA ladder. Figure S5. Phenotype of WT and fad2–2 mutant tobacco seed. a, Mature seeds from WT and fad2–2 mutant lines. Bar = 500 μm. b, Average seed length. Data are mean ± SD (n = 30). c, Average seed width. Data are mean ± SD (n = 30). d, Average seed weight. Values are mean ± SD of five individual measurements of 50 seeds/replicate. [file 12870_2020_2441_MOESM1_ESM.doc]

**Additional file 1**


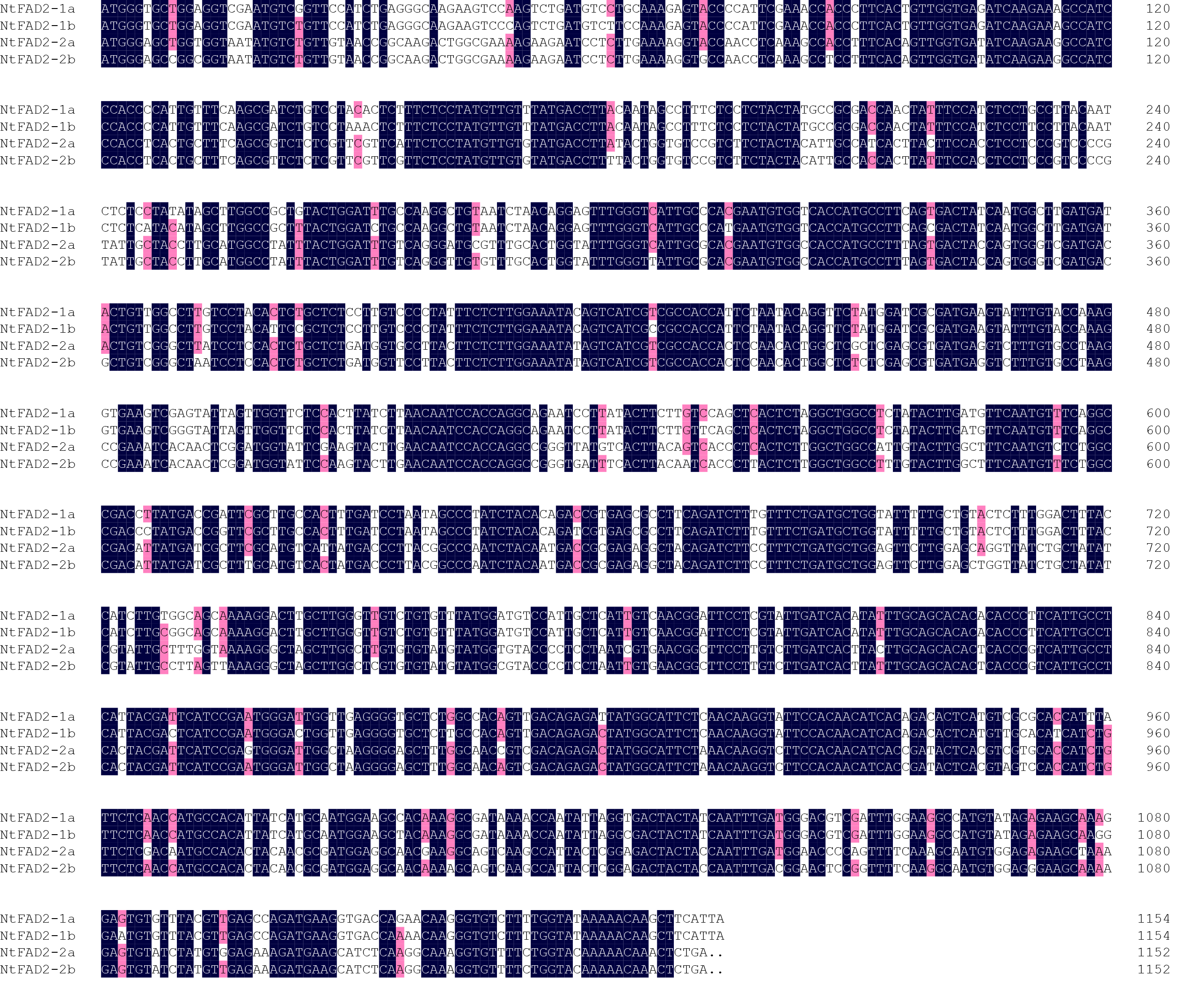


**Figure S1.** Protein coding sequence alignment between *NtFAD2-1a*, *NtFAD2-1b*, *NtFAD2-2a*, and *NtFAD2-2b*.


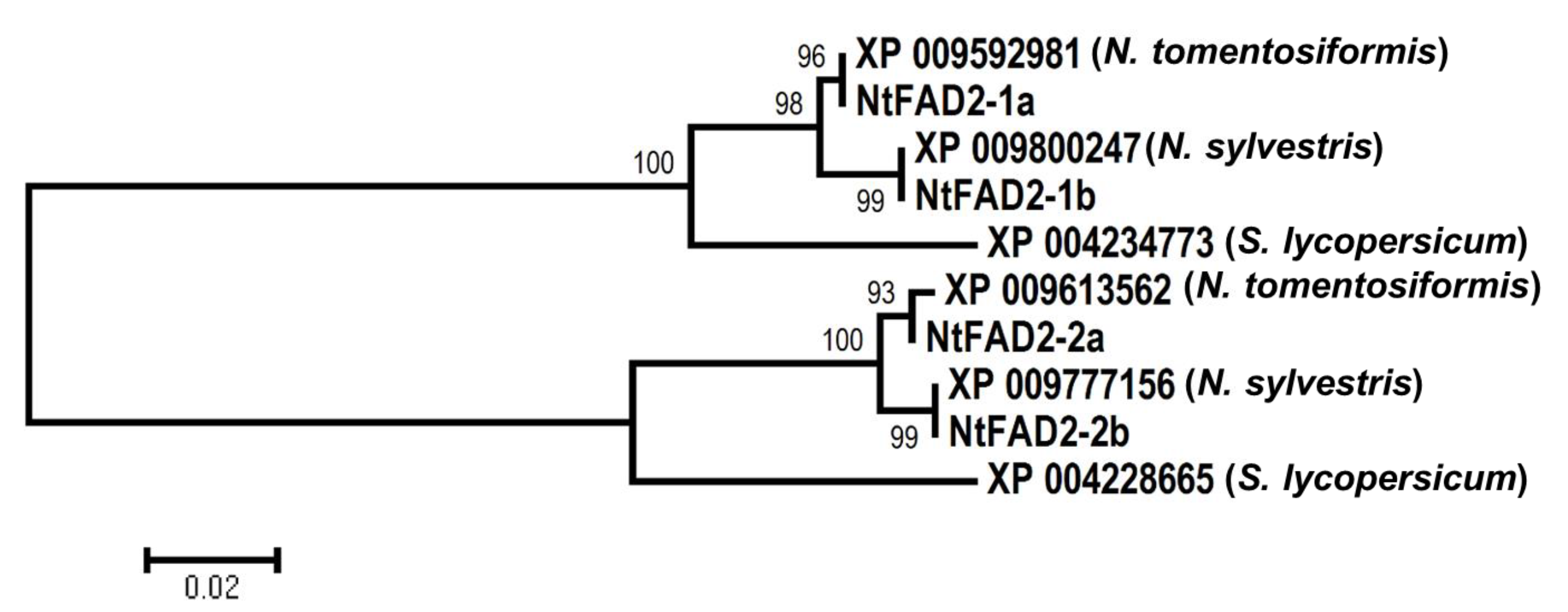


**Figure S2.** Phylogenetic analysis of FAD2 proteins from *N. tabacum*, *N. tomentosiformis, N. sylvestris, and S. lycopersicum*.


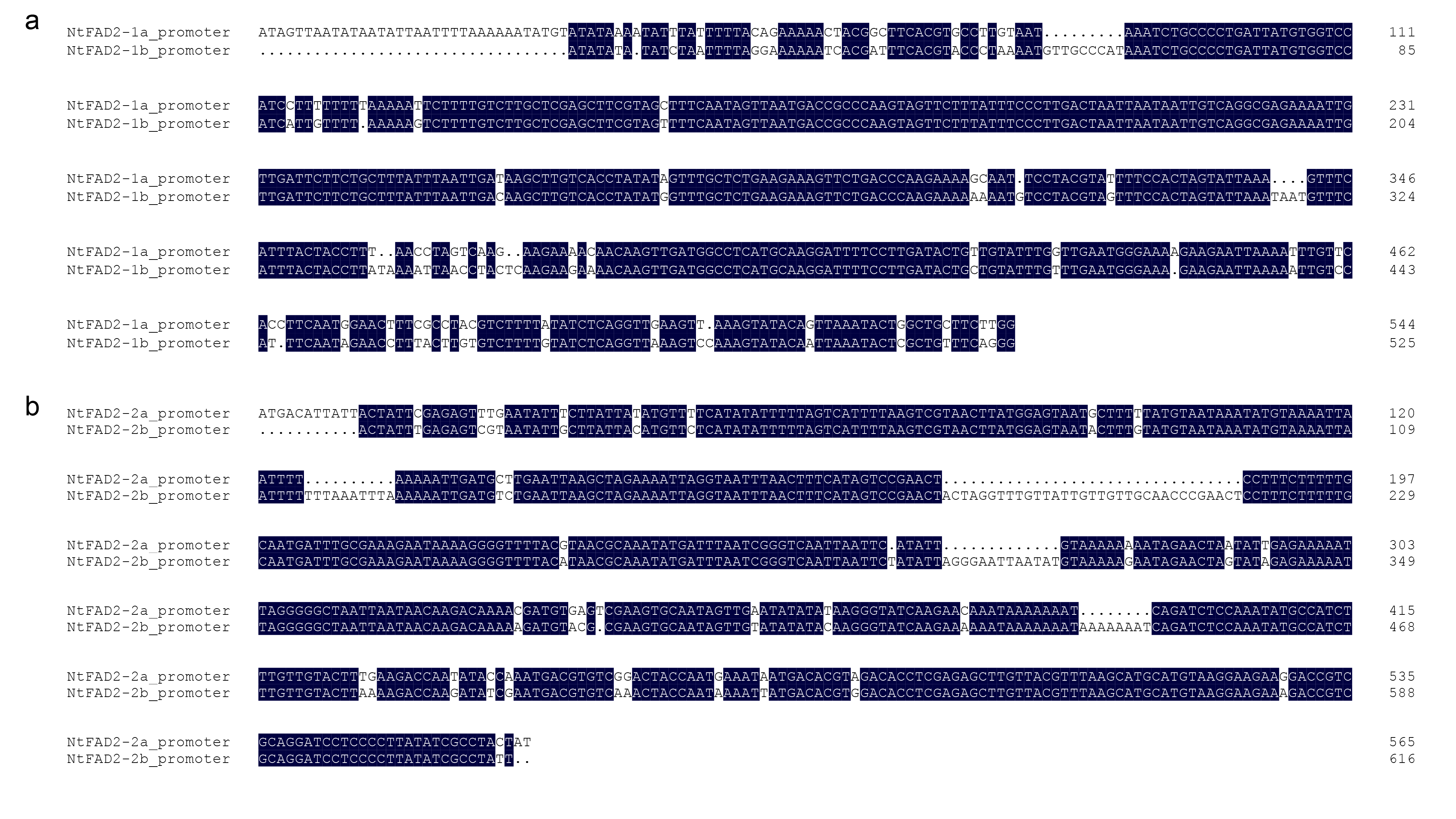


**Figure S3.** Alignment of the *NtFAD2* genes promoter sequence. a. Alignment of promoter sequence of *NtFAD2-1a* and *NtFAD2-1b*. b. Alignment of promoter sequence *NtFAD2-2a*and *NtFAD2-2b*.


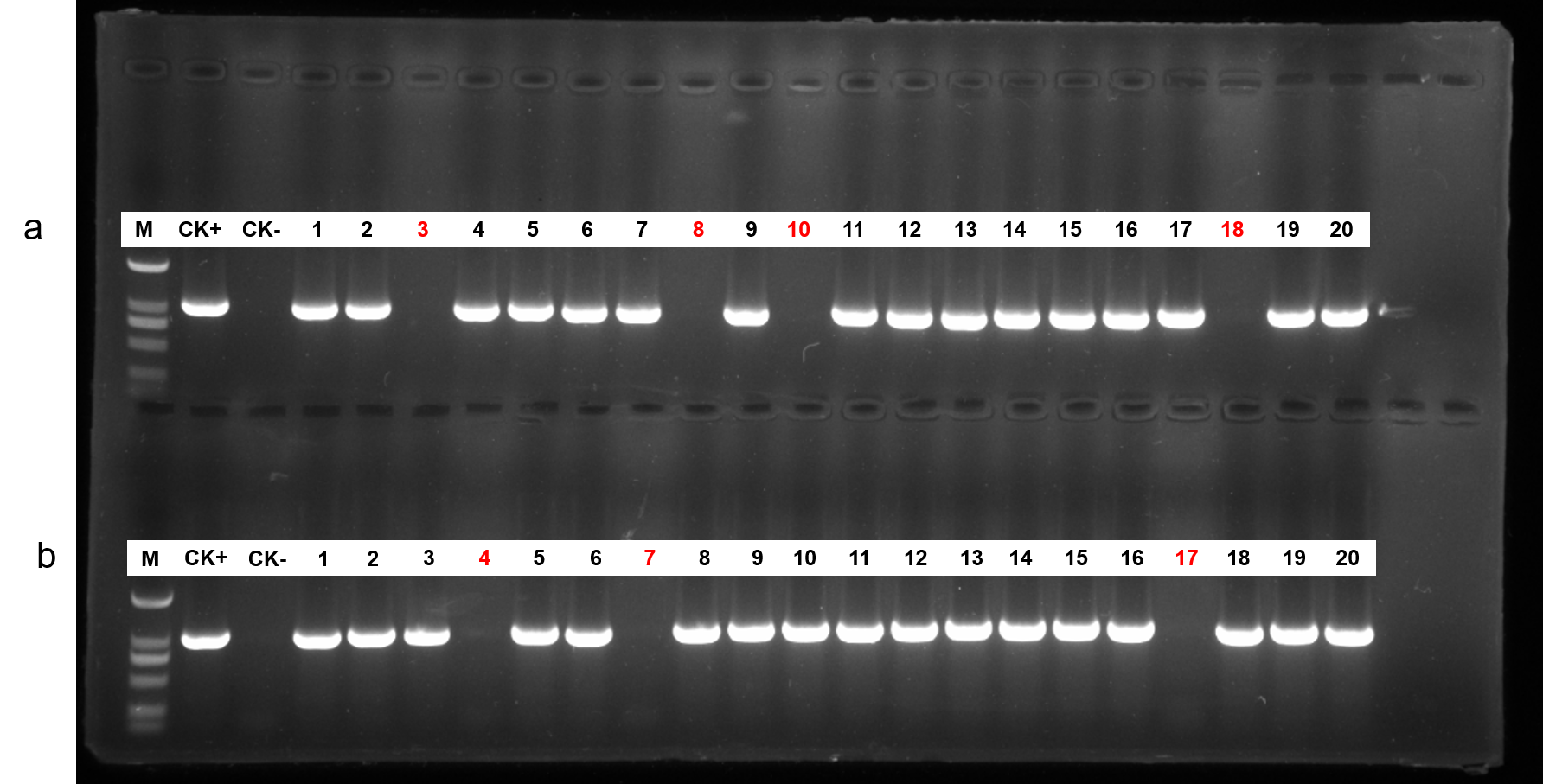


**Figure S4.** PCR-based identification of T-DNA-free T1 segregants using primers directed at the Cas9 gene sequence. Segregants among (**a**) *fad2-2#06* plants, and (**b**) *fad2-2#14* plants. The vector pKSE401-FAD2-2 was used as the positive control (CK+) and DNA from WT plant as the negative control (CK-). M: DNA ladder.


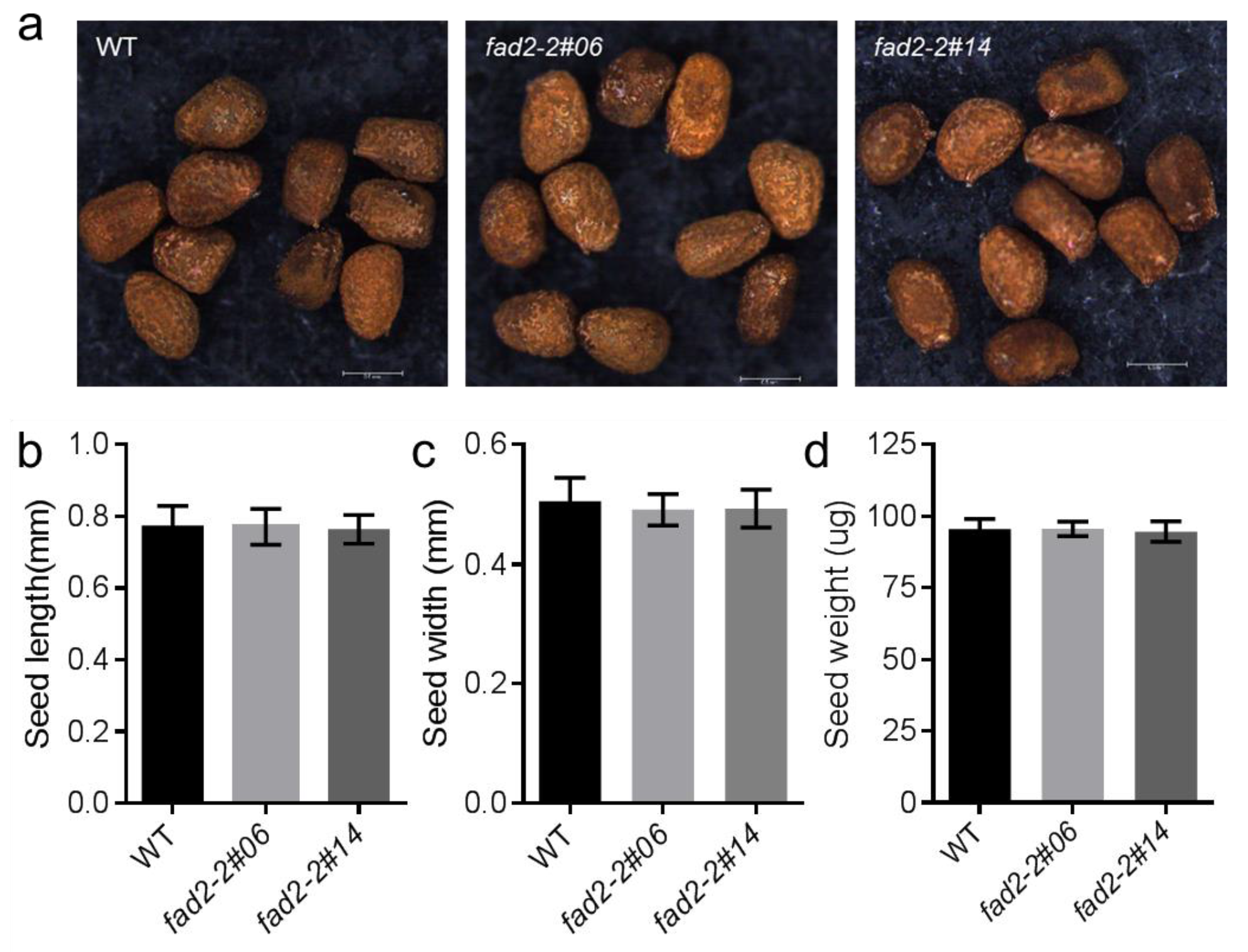


**Figure S5.** Phenotype of WT and *fad2-2* mutant tobacco seed. **a**, Mature seeds from WT and *fad2-2* mutant lines. Bar = 500 μm. **b**, Average seed length. Data are mean ± SD (n = 30). **c**, Average seed width. Data are mean ± SD (n = 30). **d**, Average seed weight. Values are mean ± SD of five individual measurements of 50 seeds/replicate.
