# Supplementary material for: Design of high-oleic tobacco (Nicotiana tabacum L.) seed oil by CRISPR-Cas9-mediated knockout of NtFAD2–2
Source: BMC Plant Biol. 2020 May 25;20:233. doi: 10.1186/s12870-020-02441-0 (PMC7249356; doi:10.1186/s12870-020-02441-0)
Supplement: Supplementary file 2 — Additional file 2 Table S1. Fatty acid profile of yeast cells transformed with NtFAD2–2 genes. Table S2. List of primers used in this study. [file 12870_2020_2441_MOESM2_ESM.doc]

**Additional file 2**

**Table S1. Fatty acid profile of yeast cells transformed with *NtFAD2-2* genes**

| Vector | Fatty acid composition (mol%) | | | | | |
| --- | --- | --- | --- | --- | --- | --- |
|  | 16:0 | 16:1^Δ9^ | 16:2^Δ9, 12^ | 18:0 | 18:1^Δ9^ | 18:2^Δ9, 12^ |
| Empty vector | 28.7±0.4 | 44.4±3.2 | 0±0.0 | 4.8±0.1 | 22.1±0.5 | 0±0 |
| pDR195:NtFAD2-2a | 27.3±0.7 | 32.5±2.9 | 9.5±0.2 | 7.7±0.2 | 10.1±0.2 | 13.1±0.1 |
| pDR195:NtFAD2-2b | 26.4±0.6 | 33.26±2.3 | 8.8±0.1 | 8.3±0.1 | 12.1±0.3 | 11.1±0.2 |

16:0, palmitic acid; 16:1^Δ9^, palmitoleic acid; 16:2^Δ9, 12^, hexadecadienoic acid; 18:0, stearic acid; 18:1^Δ9^, oleic acid; 18:2^Δ9, 12^, linoleic acid. The data are the mean ± SD of three independent experiments.

Table S2. List of primers used in this study

| Primer name | Sequence (5’-3’) | Used for |
| --- | --- | --- |
| qNtFAD2-1a-F | TAACATTTACATATATAGAGAGAG | qRT-PCR |
| qNtFAD2-1a-R | CGAATGGGGTACTCTTTGCAG |
| qNtFAD2-1b-F | CAAATAACATTCACATAGGGAGAG |
| qNtFAD2-1b-R | GAATGGGGTACTCTTTGGAAG |
| qNtFAD2-2a-F | GAGGTTTGGGATTGACGAGGC |
| qNtFAD2-2a-R | GAATGAACGAACGAGAGACCGCT |
| qNtFAD2-2b-F | TGTGTGTGGGGGTGAAGGCTC |
| qNtFAD2-2b-R | GAGAACGAACGAACGAGAGAAC |
| pDR195-NtFAD2-2a-F | AAAATATACCCCAGCCTCGAGATGGGAGCTGGTGGTAATATG | Yeast expression vector construction |
| pDR195-NtFAD2-2a-R | AAGAAGTCCAAAGCTGGATCCTCAGAGTTTGTTTTTGTACCAG |
| pDR195-NtFAD2-2b-F | AAAATATACCCCAGCCTCGAGATGGGAGCCGGCGGTAATATGTC |
| pDR195-NtFAD2-2b-R | AAGAAGTCCAAAGCTGGATCCTCAGAGTTTGTTTTTGTACCAG |
| pKSE-NtFAD2-2-F | ATTGTGAAAGCAGTGAGGTGGGA | CRISPR/Cas9 vector construction |
| pKSE-NtFAD2-2-R | AAACTCCCACCTCACTGCTTTCA |
| ge-NtFAD2-2-F | ACTGGCGAAAAGAAGAATCCTC | Target flanking sequence amplification |
| ge-NtFAD2-2-R | GTTGGAGTGGTGGCGACGATGAC |
| Cas9-F | GACCACGACGGGGATTACAAGG | *Cas9* gene fragment amplification |
| Cas9-R | GGTGTTCACCCTGAGAATATCAG |
